# Supplementary material for: The Use of PediSTAT Application by Paramedics Working in Saudi Arabia to Reduce the Risk of Medication Error for Pediatric Patients
Source: Pediatr Rep. 2025 Jan 16;17(1):9. doi: 10.3390/pediatric17010009 (PMC11755567; doi:10.3390/pediatric17010009)
Supplement: Supplementary file 1 [file pediatrrep-17-00009-s001.zip › pediatrrep-3381200-supplementary.pdf]

SUPPLEMNETARY FILE S1: STUDY QUESTIONNAIRE

Data Collection Sheet

Personal Information	
Age	__________
Gender	Male<br>Female
Degree	Diploma<br>Bachelor<br>Master<br>PhD
If you have a Master or PhD degree, what program have you taken the degree in?	__________
Workplace	MNGHA<br>SRCA<br>Other: ______
Do you have a certification in pediatric courses?	Yes<br>No
If yes, what is the course?	__________

What is the method that the paramedic will use?

- o Conventional method
- o Pedi STAT application

**Scenario 1 (Cardiac Arrest)**	
You are dispatched to a 9-year-old male after a drowning accident. His mother dragged him out and noticed the absence of breathing and pulse, so she had started chest compressions. You have arrived at the scene after 4 minutes of CPR. Your partner has taken over the chest compressions, and you immediately have attached the monitor and the defibrillator pads. No intravenous access can be opened, so you have opened an intraosseous line. You checked the rhythm, but it was an asystolic rhythm, so you have decided to give him Epinephrine. Thepatient weighs 36 kg.	
Dose	
Route	

**Scenario 2 (Severe Asthma)**	
You have arrived at a scene where you find a 10-year-old female who has difficulty breathing.She is unable to speak, her lips are blue, and you have noticed audible wheezing. Immediately,you have attached the monitor. Vital signs: SpO2: 89%, HR: 123, BP: 135/90Pateint approximately weighs 40 Kg	
What is the treatment of choice?	
Dose	
Route	

**Scenario 3 (Seizure)**	
You are dispatched to an 8-year-old male whose father stated that his son was playing video games, and suddenly he has been starting shaking. When you have arrived at the scene, patientis actively seizing. You have checked his blood glucose level, and it is normal, so you decide to give him Midazolam. The patient weighs 32 kg.	
Dose	
Route	

Scenario 4 (Hypoglycemia)	
You are dispatched to an 11-year-old male who is known of having type 1 diabetes. His mother told you that he didn't eat his breakfast this morning, and he suddenly collapsed. Younotice that he is suffering of SOB and dizziness.Vital signs: RR: 28, HR: 130, BP: 110/60, SpO2: 91%, BGL: 34mg/dL (1.2 mmol/L).You start supporting him with oxygen and establishing an IV access. Patient approximately weighs 43 kg.	
What is the treatment of choice?	
Dose	
Route	

SUPPLEMENTARY FILE S2: Pedi STAT DEFINITION AND EXPLNATION

PediSTAT is a comprehensive smartphone application designed to serve as a high-quality point-of-care tool for emergency professionals, specifically focused on pediatric care. The app provides quick and reliable access to medication dosages and equipment sizes based on pediatric weight, helping streamline emergency decision-making.

Dr. James Kemper, a Fellow of the American College of Emergency Physicians, with contributions from a team of emergency physicians, PharmDs, and software developers, created the content of PediSTAT. The software is licensed and subject to terms and conditions, with regular updates and revisions provided by the owner to ensure that it remains current and accurate. The study obtained permission from the developer to use it for research purposes.

Medication dosages, concentrations, and availability within the app are sourced from trusted references such as Micromedex, The Cochrane Collection/Cochrane Reviews, Medscape, and UpToDate (Wolters Kluwer). Additionally, specialized references were used for sections like Airway Management, Anaphylaxis, and Cardiac Resuscitation.

One of the critical features of PediSTAT is its ability to calculate medication dosages based on a child's weight (in both kg and lbs), age, and height (in both cm and inches). The app's developers have made extensive efforts to ensure the accuracy of dosage calculations and equipment recommendations, offering healthcare providers a reliable tool for pediatric emergency care.

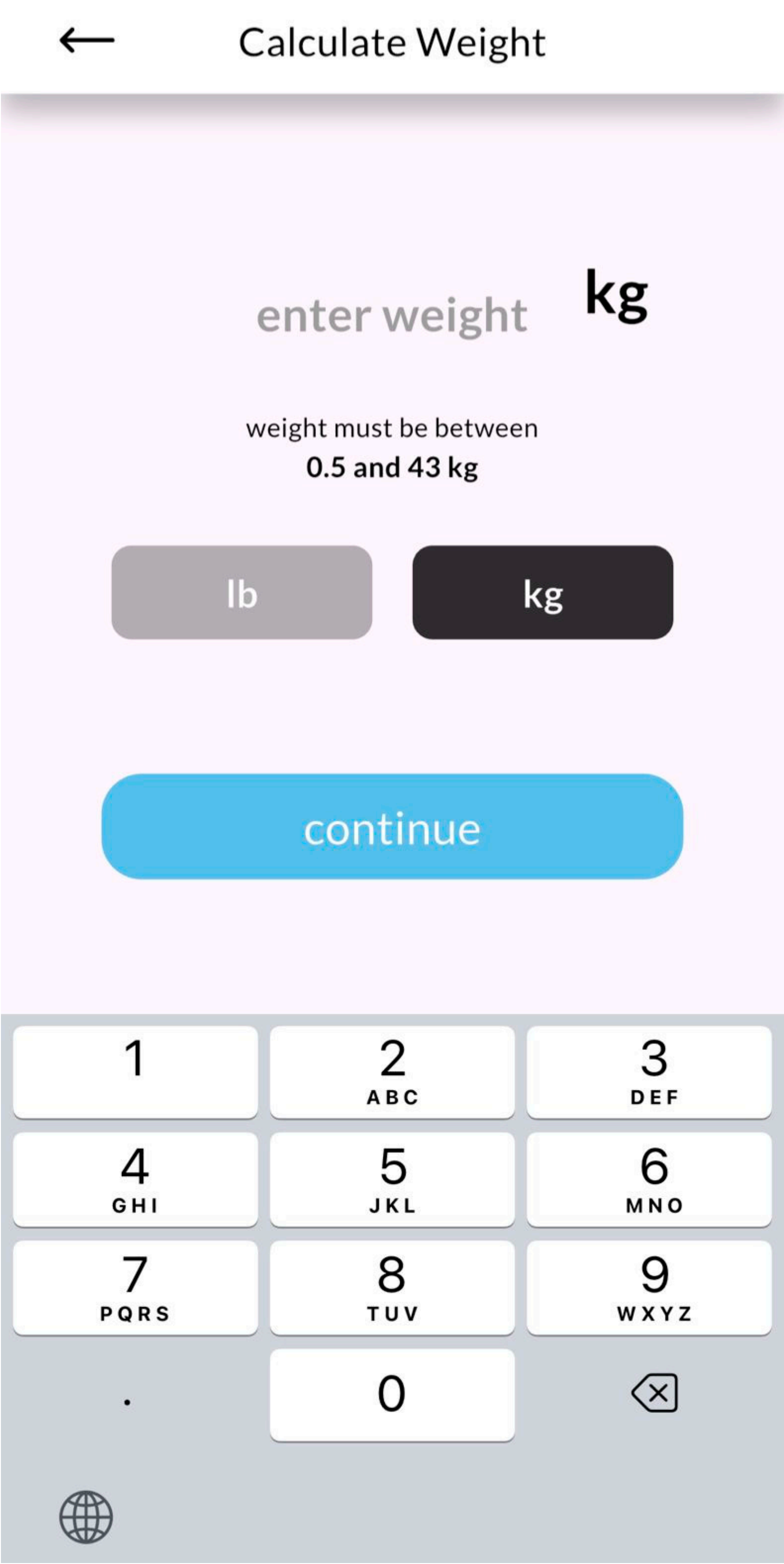
Calculate Weight
enter weight
kg
weight must be between
0.5 and 43 kg
lb
kg
continue
1
2
ABC
3
DEF
4
GHI
5
JKL
6
MNO
7
PQRS
8
TUV
9
WXYZ
.
0

← Categories References

categories, medications, equipment, etc

- Airway Intervention / Intubation
- Anaphylaxis / Allergic Reaction
- Antiemetics
- Antimicrobials (IV/IM)
- Burns
- Cardiac Resuscitation
- Equipment
- Fever
- Fluid/ Blood Resuscitation

30.0 kg | 7y 6m | 131.5 cm

← Categories *References*

30.0 kg | 7y 6m | 131.5 cm

Cardiac Resuscitation / General Dosing

CPR

Compressions:

100-120 Compressions / minute
Rhythm / Pulse check every 2 minutes

Respirations:

20-30 Breaths / minute
Goal tidal volume 6 mL/kg

Tidal Volume (goal) 180 mL

Epinephrine 0.1 mg/mL (1:10,000) IV/IO [+]

Epinephrine 1 mg/mL (1:1000) Endotracheal [+]

Adenosine IV/IO [+]
(Adenocard, Adenoscan)

Amiodarone IV/IO [+]
(Pacerone, Cordarone, Nexterone)
